# Viral immunogenic footprints conferring T cell cross-protection to SARS-CoV-2 and its variants

**DOI:** 10.3389/fimmu.2022.931372

**Published:** 2022-07-28

**Authors:** Eduardo Cheuiche Antonio, Mariana Rost Meireles, Marcelo Alves de Souza Bragatte, Gustavo Fioravanti Vieira

**Affiliations:** ^1^ Post Graduation Program in Genetics and Molecular Biology, Universidade Federal do Rio Grande do Sul, Porto Alegre, Brazil; ^2^ Post Graduation Program in Health and Human Development, Universidade La Salle Canoas, Canoas, Brazil

**Keywords:** T-cell epitopes, structural analysis, cross-reactivity, cellular response, immuno informatics, SARS-CoV-2, Coronaviruses

## Abstract

COVID-19 brought scenes from sci-fi movies into real life. Infected individuals include asymptomatic cases to severe disease leading to death, suggesting the involvement of the genetic constitution of populations and pathogens contributing to differential individuals’ outcomes. To investigate shared immunogenic features between SARS-CoV-2 targets and other coronaviruses, we modeled their peptides in 3D structures of HLA-A*02:01 (pMHC), comparing their molecular surfaces These structures were also compared with a panel of epitopes from unrelated viruses, looking for potential triggers conferring cross-protection in uninfected individuals. As expected, SARS-CoV 1 and 2 peptides share molecular and physicochemical features, providing an explanation for the verified experimental immunogenicity among them. Surprisingly, even discordant sequences from human coronaviruses 229E, OC43 and epitopes from unrelated viruses involved in endemic human infections exhibit similar fingerprints of immunogenicity with SARS-CoV-2 peptides. The same approach indicates a conserved CD8+ T cell recognition between Wuhan SARS-CoV-2 sequences and altered peptides from Variants of Concern. Examination of structural data over epitope sequence analysis here could explain how previous infections may produce a heterologous immunity response in a global scale against emergent diseases such as Covid-19, mitigating its full lethal potential, and paves the way for the development of wide spectrum vaccine development.

## Introduction

COVID-19 has brought scenes from sci-fi movies into real life. A new virus that spreads in a worldwide fashion, leaving a trail of deaths and fear of the unknown, especially in the beginning. The understanding of all involved aspects in SARS-Cov-2 pathogenesis and mechanisms of immune system elicitation is a crucial step to fighting out the current pandemics and preventing future tragedies. It is important to recognize the SARS-CoV-2 particularities, which can be initially achieved by the comparison with its relatives. The coronaviridae family includes other respiratory syndrome causative agents in humans, like the SARS-CoV and MERS-CoV viruses (1).

In the current pandemic, a growing amount of evidence demonstrates the importance of cellular responses in the SARS-CoV-2 viral clearance (2). Chen et al. (3) presented several works where severe patients exhibited lymphopenia, especially for CD8+ T cells, compared with mild patients or healthy controls. Besides, the cytotoxic T cells in individuals with severe symptoms have diminished levels of cytokine secretion, as YFN-gamma. In order to properly clear the SARS-CoV-2 infection, both arms of immune response must be triggered, and act in a coordinated way, to properly clear the SARS-CoV-2 infection (4–6). So, in addition to the production of neutralizing antibodies, CD4+ and CD8+ T cell responses should be activated (7, 8). These elements include a set of proteins, such as proteasome subunits, TAP1/2 and MHC, orchestrating cellular immune responses as central players. Most of their genes belong to the MHC locus, the most polymorphic human genomic region (9), and this variability enables animal species to face the mutational potential of viruses. Such an effect can be more evident at a populational level. Briefly, these proteins are involved with the processing and presentation of small peptides in cell membranes, allowing the immune system the discrimination of self from non-self, thus eliminating pathogens and tumors. At this point, a critical question arises: not only the MHC genes are involved in the immune response, but also other proteins (TAP, proteasome subunits, and proteins from Peptide Loading Complex), fundamental to producing true epitopes, belong to this genomic region. Many works, aiming to prospect tumoral or vaccine targets, focus their predictions only on the ligandome from proteins of pathogens or cancer samples (10). A potential to bind to different MHC alleles does not confer to peptides their full potential to be a T cell epitope. The complete triggering of a T cell synapsis demands additional requirements, involving epitope immunodominance and pMHC : TCR physicochemical complementarity. Thus, *in silico* analysis considering additional steps on the antigen processing pathway and comparisons among putative targets and immunogenic epitopes, could present a better performance to prospect actual T cell epitopes, as in the current situation where no previous information is available.

It is known that different HLA alleles can bind and present the same viral target (peptides) with altered efficiencies, which could provoke both susceptibility or resistance to a disease caused by a specific pathogen, depending on the type of MHC that the individual possesses (11). Thus, some HLA allotypes are unable to present some recognized immunodominant epitopes, which could impair the efficient immune response triggering. The opposite also occurs, the existence of HLAs with improved potential to present optimal viral targets, allowing infection control (12). The work of Agerer et al. (13) already demonstrates that SARS-CoV-2 mutations in MHC-I-restricted epitopes could evade CD8+ T cell responses by altering the anchor residues that provide stability. It is important to notice that not only the MHC binding is important to immunogenicity, but also the region of interaction with TCR in pMHC complexes provides information able to elicit a T cell effector response, with a percentile of these cells that will constitute the memory cells reservoir. In this sense, it is interesting to investigate immunogenic footprints left by previous infections, searching for cross-reactive targets with SARS-CoV-2 epitopes. Probably, those targets are recognized by the memory T cells pool from the world population individuals’, which could be granting a buffering effect, avoiding a more lethal pandemic. The importance of cross-reactivity in Covid-19 is discussed in the work of Saletti et al. (14). They observed that older adults lack SARS-CoV-2 cross-reactive T lymphocytes directed to human coronaviruses, OC43 and NL63, which could be a partial explanation for the more severe clinical outcome observed in this group.

### HLA-A*02:01 as an example of universal protective allele in human populations

**Figure 1:**
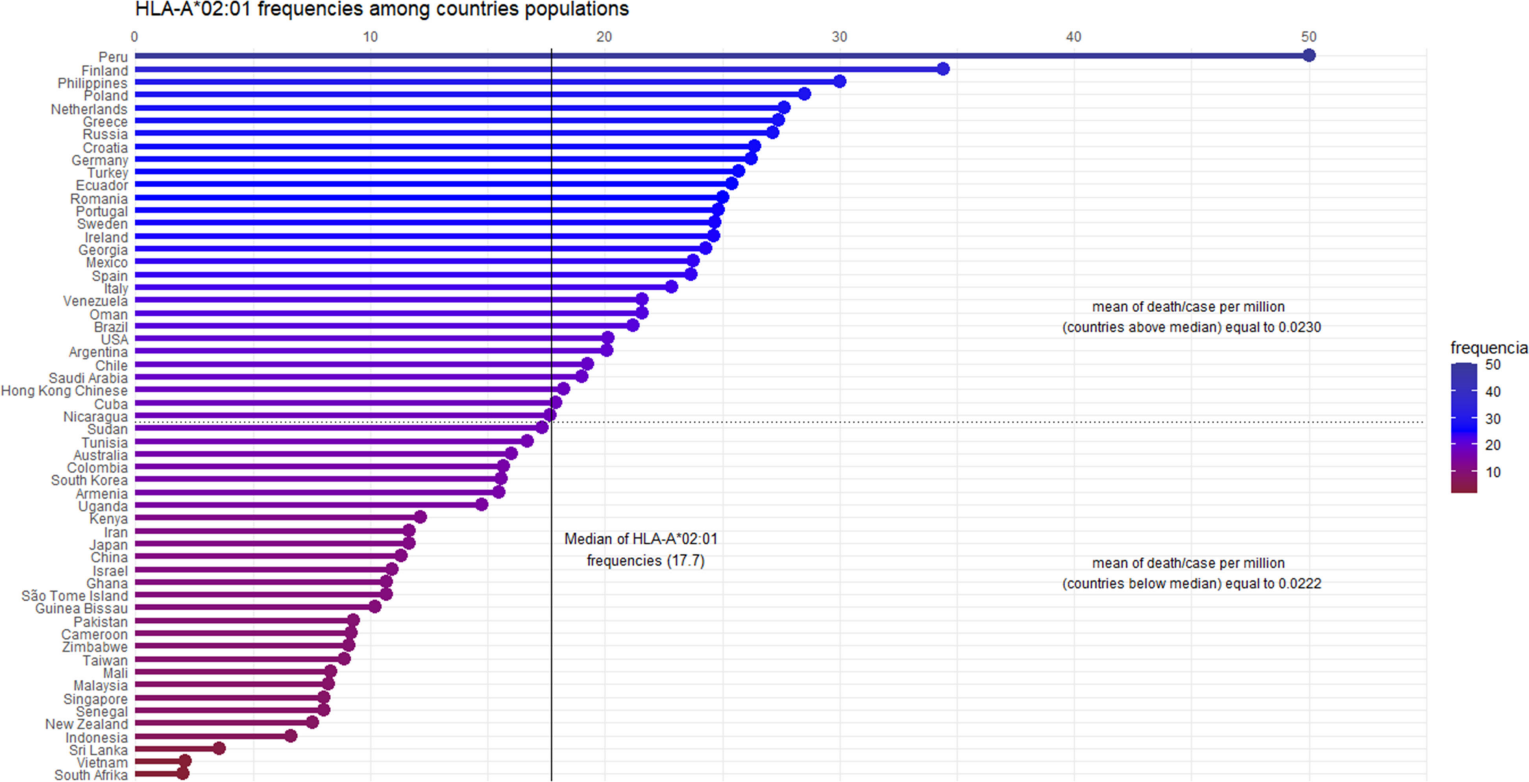
Graphical representation of the HLA-A*02:01 allele frequencies, by country. Fifty-seven countries are ordered by their allele frequencies (X-axis) from the highest to the lowest. The frequency values were calculated based on Alleles Frequency Net Database, filtering for gold and silver standard samples. Considering more than one population per country, we estimated the weighted average to represent countries’ frequency. The median of HLA-A*02:01 frequency among the 57 countries is 17.7. Ninety-five percent of the countries have frequency values above 5%, showing the high allele prevalence among the human populations. The death/case per million average shows no statistical difference (confidence intervals of 95 and 99 percent) among countries above and below the median (p - value = 0.8481).

We hypothesized that pre-existing T cell responses against SARS-CoV-2 are through prevalent alleles in populations, such as the HLA-A*02 supertype. Migliorini et al. (2021) revised the literature and found some works where HLA-A*02:01 was associated with a risk of infection and worse prognosis in some populations. Initially, it seems to be contradictory reasoning, but considering mortality rates in other SARS causative viruses (around 10% in SARS-CoV-1 and 35% in MERS) (15, 16), the current values (slightly above 2%) may indicate a more effective worldwide cellular response. The idea of using HLA-A*02:01 as a model is supported by many studies reporting it as a pivotal allele in viral infections. A consultation on Immune Epitope Database (www.iedb.org) returned 896 references describing positive T cell response against viral peptides presented in the context of the HLA-A*02:01 allele (accessed on March 6, 2021). Besides, 152 out of 1146 immunogenic epitopes described for coronaviruses are restricted to HLA-A*02:01. During the SARS outbreak of 2003, lymphocytes from previously infected individuals were able to eliminate cells presenting SARS-CoV epitopes restricted to HLA-A*02:01 molecules up to six years later from recovering (17–19), demonstrating the importance of this allele on viral clearance and T cell central memory. A conducted study with individuals from China and Hong Kong evidenced that more than half of the SARS recovered subjects were HLA-A*02:01 positive (20). In the present pandemic Italian individuals harboring haplotypes containing the HLA-A*02:01 allele presented a negative correlation between incidence and death (21). In another work, a risk score was developed by Shkurnikov et al., 2021 aiming to predict the possibility of an individual to present severe acute respiratory syndrome coronavirus 2, associated with the presence of the HLA-A*02:01 allele with low risk (22). In Spanish patients, this allele was overrepresented in mild patients, suggesting its involvement in a better prognosis of the disease (23, 24). It is noteworthy considering its high-prevalence among human populations, with frequencies among 57 analyzed countries presenting a median of about 17.7 (**Figure 1**). While we cannot assign its ability to confer protection, independently, by the above-presented value, this ubiquitous feature enables it as one of the main tools in the proposed global shield.

**Figure 2:**
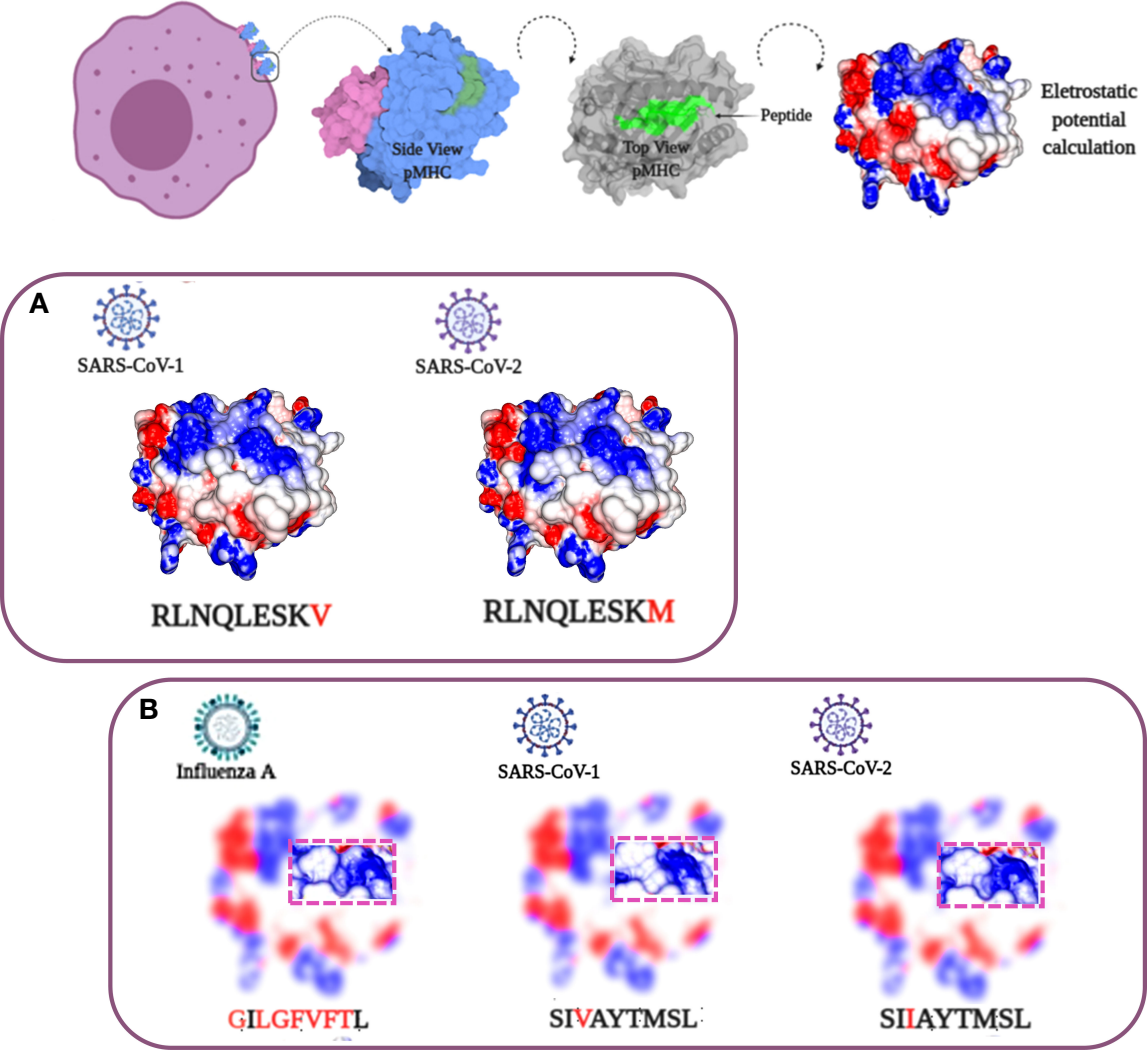
Structural analysis of SARS-CoV HLA-A*02:01 epitopes. At the top, a progressive scheme demonstrating the analyzed TCR-interacting surface. From left to right: 1) the MHCs in the cell membrane; 2) a side view of pMHC with alpha chain in pale blue, B2-microglobulin in pale purple, and the peptide in green; 3) a top view of pMHC with the peptide region highlighted in green, and 4) the pMHC surface with electrostatic potential distribution and topography, the main elements involved in immune response stimulation. Negative charge values colored in red and positive ones in blue, with neutral charges depicted in white (variation from -3 to 3 passing through zero). **(A)** SARS-CoV peptide sequences showing high similarity in sequence and molecular features. **(B)** SARS-CoV SI**V**AYTMSL/SI**I**AYTMSL sequences presenting subtle differences. A structural comparison with an unrelated influenza IAV GILGFVFTL epitope demonstrates an even greater molecular identity with the SI**I**AYTMSL SARS-CoV-2 peptide compared with its SARS-CoV-1 cognate sequence. The image of the cel in the top was Created with BioRender.com.

In our analysis, most of the recovered HLA-A*02:01 epitopes described in past epidemics seem to present conserved sequences compared to their equivalent in SARS-CoV-2 (**Table 1**), as described in other works  (25, 26). Nevertheless, in the case of discordant peptide sequences, the simple sequence comparison of SARS-CoV-1 and SARS-CoV-2 may provide us with little information about the impact of these alterations concerning the immunogenic potential on current putative viral targets. We could not state that the immunogenicity is preserved or lost, just looking at these linear alterations. Stervbo et al., suggested that previous immunity to specific SARS-CoV-2 proteins is not driven by near-identical epitopes (27). Thus, the analysis of structural and physicochemical features in the peptide-MHC (pMHC) surfaces that contact the T cell receptors may provide us with additional information involved in T cell activation. Such structural investigation has already demonstrated its potential, explaining differential immunogenicity among epitopes from diverse viral strains or tumoral origins (28, 29). Since there is no available crystal for all specific targets (peptides) complexed in HLA-A*02:01 allele, we constructed all customized complexes through our reliable DockTope tool for pMHC modeling (http://tools.iedb.org/docktope/) (30). This structural analysis gives us two alternative scenarios: the TCR interacting surfaces of both pHLA-A*02:01 complexes were similar (**Figure 2A**), explaining the preserved immunogenicity observed in 19 out 20 SARS-CoV-2 discordant sequence targets (except for ILPDPSKPS, with no positive T cell assay in SARS-CoV-2 – **Table 1**) (**Figure S1**); or the targets presented subtle physicochemical alterations in complexes harboring SARS-CoV-2 peptides, compared to former SARS-CoV. Amazingly, in this case, some of them turned into closely related surfaces from previously described immunogenic epitopes from non-related viruses (**Figure 2B**). In the depicted example, the SARS-CoV-2 SIIAYTMSL peptide demonstrates structural convergence of physicochemical features with the immunodominant Influenza virus epitope M1_58-66_ GILGFVFTL. Both epitopes share 2/9 amino acids, reinforcing the importance of structural investigation over sequence comparison to prospect cross-reactive targets. An impressive point is that other work using analysis of specific TCR sequences for SARS-CoV-2 had a known specificity for the Influenza virus M1 immunodominant epitope. Here, we present a structural basis for it (31). These first comparisons uncovered exciting scenarios. Firstly, even those peptides with sequence alterations in SARS-CoV-2, but without molecular modifications in the TCR interacting surfaces, reveals that our method is reliable to prospect good candidates for immunization strategy in new pandemic events. Moreover, some of these peptides’ sequences resemble highly immunogenic epitopes from other viral organisms, which emphasizes a necessity to investigate this new face of the immunogenic prism, that is, previous infections triggering cross-reactivity events. In our analysis, to highlight that the comparisons were not resulting from structural biases, we provide a small sample of TCR interacting surfaces from immunogenic epitopes restricted to HLA-A*02:01, from CrossTope database (www.crosstope.com) (**Figure S2**) (32).

**Table 1:** Recovered HLA-A*02:01 epitopes from SARS-CoV SARS-Cov-2 and other alpha and beta coronaviruses members.

IEDB - experimental positive epitopes from coronaviruses ^A^					Corresponding peptides in other coronaviruses ^B^			
Epitope ID	Description	Antigen Name	Organism Name	Experimental Assays (Positive/All)	SARS-CoV-2^C^	HCoV-229E	HCoV-NL63	HCoV-OC43
2801	ALNTLVKQL	S protein	SARS-related coronavirus	2/2		**S**LN**H**L**TS**QL	ALN**H**L**TS**QL	ALN**N**L**LQ**QL
2802	ALNTPKDHI	Nucleoprotein	SARS-related coronavirus	2/2		**RVTV**PKDH**P**		**DV**NTP**ADIV**
16156	FIAGLIAIV	Spike glycoprotein precursor	SARS-related coronavirus	2/2				FI**N**G**IF**A**KV**
21347	GMSRIGMEV	Nucleoprotein	SARS-related coronavirus	10/10				
27182	ILLNKHIDA	Nucleoprotein	SARS-related coronavirus	1/3				
27241	ILPDPLKPT	Spike glycoprotein precursor	SARS-related coronavirus	2/2	ILPDP**S**KP**S**			
34851	LALLLLDRL	Nucleoprotein	SARS-related coronavirus	4/4				
36724	LITGRLQSL	Spike glycoprotein precursor	SARS-related coronavirus	5/8		LITGRL**AA**L	LITGRL**AA**L	LI**N**GRL**TA**L
37473	LLLDRLNQL	Nucleoprotein	SARS-related coronavirus	11/12				
38881	LQLPQGTTL	Nucleoprotein	SARS-related coronavirus	4/6		**QK**LP**N**G**V**T**V**		**GTV**LPQG**YY**
44814	NLNESLIDL	S protein	SARS-related coronavirus	3/4		N**I**N**ST**L**V**DL		**V**LN**H**S**Y**I**N**L
54690	RLNQLESKV	Nucleoprotein	SARS-related coronavirus	9/11	RLNQLESK**M**			
58730	SIVAYTMSL	S protein	SARS-related coronavirus	3/3	SI**I**AYTMSL			
69657	VLNDILSRL	S protein	SARS-related coronavirus	3/4			**ET**ND**VS**S**M**L	**S**L**QE**ILSRL
71663	VVFLHVTYV	Spike glycoprotein precursor	SARS-related coronavirus	5/5				**LY**F**I**H**FN**YV
125100	ILLNKHID	Nucleoprotein	SARS-related coronavirus	1/1				
21041	GLMWLSYFV	Membrane glycoprotein	SARS coronavirus TJF	7/8	GLMWLSYF**I**	**LV**MW**VM**YF**A**	**LCL**W**VM**YFV	**II**MW**IV**YFV
64710	TLACFVLAAV	Membrane glycoprotein	SARS coronavirus TJF	5/5			**V**LA**LSIFDCF**V	
32069	KLPDDFMGCV	Spike glycoprotein precursor	SARS coronavirus BJ01	2/2	KLPDDF**T**GCV			**YSF**D**SYL**GCV
54680	RLNEVAKNL	Spike glycoprotein precursor	SARS coronavirus BJ01	1/1		RLN**Y**VA**LQT**	**T**L**Q**E**F**A**Q**NL	RL**Q**E**AI**K**V**L

**^A^** The selection and data of the epitopes were accessed on Immune Epitope Database and Analysis Resource (IEDB - https://www.iedb.org/ - acessed on March 2021).

^B^Comparative scheme among the selected positive epitopes from coronaviruses SARS-CoV-2 peptide and other coronaviruses. The blank cells in the columns mean there is no amino acid difference among SARS-CoV-1 epitope and its equivalent in the depicted viruses. Discrepant amino acids were bolded discrepancies were** bolded.**

**^C^** All corresponding peptides in SARS-CoV-2 were already confirmed in T cell assays except the peptide sequence ILPDP**S**KP**S.**

### Searching for SARS-CoV-2 shared immunogenic fingerprints in targets from HCoVs and other prevalent viruses in human populations

**Figure 3:**
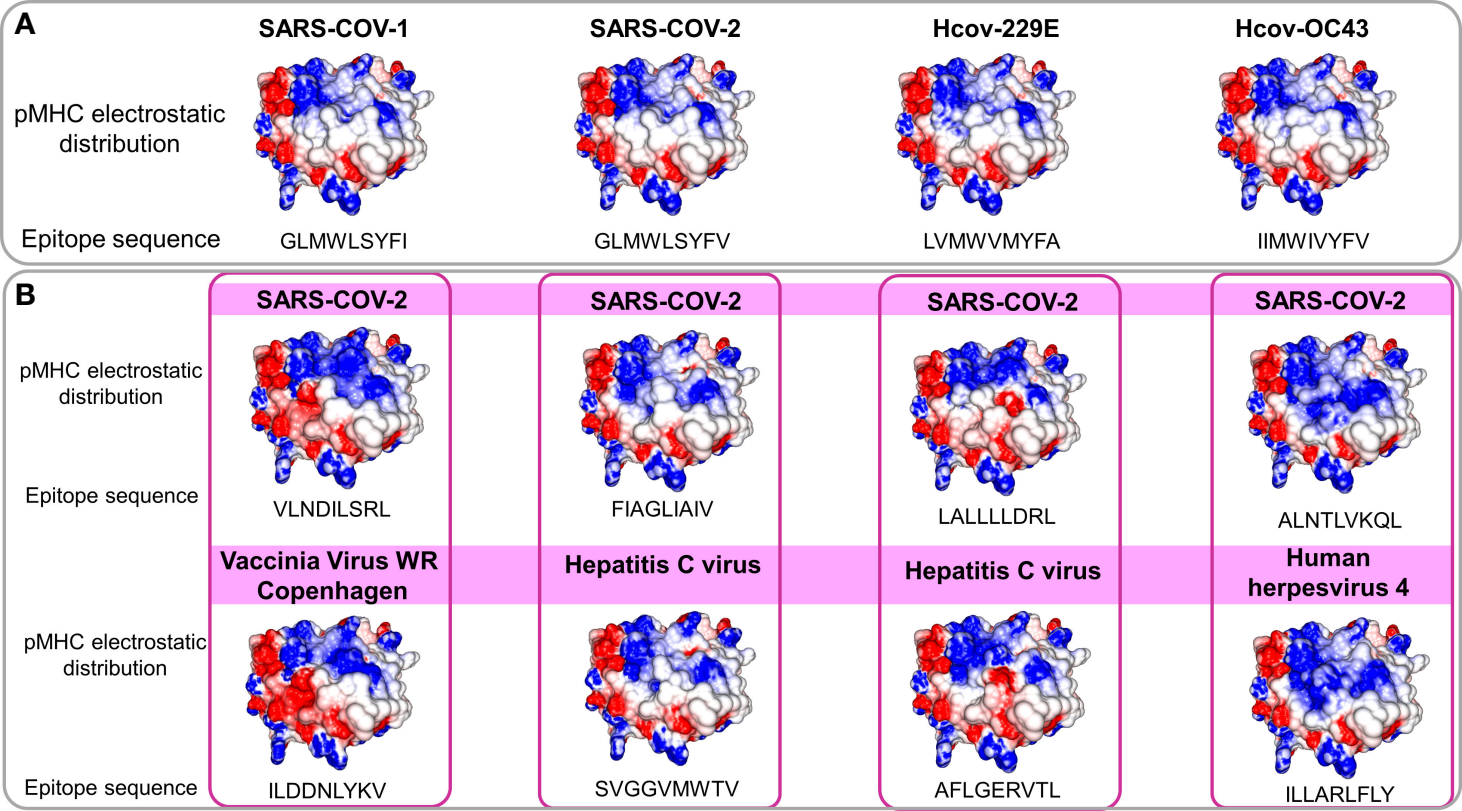
Comparison of electrostatic surface distribution and topography of SARS-CoV peptides with alphacoronaviruses and other prevalent viruses in human populations. pMHCs models compared in terms of topography and electrostatic distribution. In **(A)**, two beta (SARS-CoV-1 and 2) and two alpha and beta (229E and OC43), coronaviruses show shared electrostatic distribution and topography despite their sequence divergences. In **(B)**, a panel of four SARS-CoV peptides (top) compared with pMHCs containing viral epitopes from members of other viral families (bottom). Disregarding the lack of sequence identity and phylogenetic relationship, models present structural similarities. Electrostatic calculations are depicted as negative (red) and positive (blue) charges.

The observed molecular similarity between pMHCs complexes containing peptides from SARS-CoV-2 and Influenza virus brings us to another attractive hypothesis that refers to a universal previous cytotoxic response present in populations from all over the world, triggered by previous infections. The first suspects in the investigation were past contact with targets from remaining betacorononavirus (OC43) and alphacoronavirus genus members (229E and NL63). Epidemiological studies reported that 15-30% of the common cold events are caused by this group of pathogens (33). Even considering that they are viruses with a zoonotic origin, we would expect many spillover events throughout the history of humans, maintaining regular contact with our species (34). A codon usage analysis, involving BCoV and HCoV-OC43, suggests that an ancestor coronavirus could be present even 200 kyr ago, in early people (35). Therefore, we would expect that this group of pathogens has also contributed to shaping our current immune system repertoire. Guided by this supposition, we compared the immunogenic SARS-CoV epitopes with 229E, OC43, and NL63 corresponding protein sequences, looking for shared elements involved in immunogenicity triggering. Such analysis presented a clear example where sequence comparison might be hiding shared patterns not detectable by single amino acid identity alignment. In **Table 1**, the sequence identities ranged around 50%, a value usually not reaching thresholds of detection by regular alignment methods prospection. Nevertheless, when we inspect these same epitopes in the context of pMHC structural models harboring these peptides sequences from SARS-CoV-1, SARS-CoV-2, and alpha and betacoronavirus in HLA-A*02:01 alleles, an intriguing fingerprint arose. A similar electrostatic distribution and topography on the TCR interacting surfaces from the pMHCs, can be observed among SARS-1, SARS-2, and other coronaviruses members (229E and OC43) (**Figure 3A**). It is important to reinforce that both 229E and OC43 putative epitopes were predicted as strong binders to HLA-A*02:01 (data not shown), strengthening their potential as actual triggers for SARS-CoV cross-reactivity. The peptides from the SARS-CoV-1 and 2 are very similar (**GLMWLSYF**L and **GLMWLSYF**V). However, the corresponding peptides from 229E (LV**MW**VM**YF**A) and OC43 (II**MW**IV**YF**V) are pretty divergent (4/9 compared with the same SARS peptides), evidencing the importance of the structural investigation. Furthermore, other peptides derived from alpha-CoV viruses presented a less prominent but still interesting similarity with immunogenic SARS-CoV-1 epitopes (data not shown). However, they are also potential targets to investigate. A work recently showed that 35% SARS-CoV-2 of seronegative healthy individuals presented S-reactive CD4+ T cells. These cells react almost exclusively with the C-term epitopes region, characterized by higher similarity with spike protein of human endemic common cold coronaviruses. Nevertheless, none of the putative cross-reactive epitopes are pointed out, nor the structural basis hypothesized, reinforcing our propositions (36). Evidence of many CD4+/CD8+ cross responses against many SARS-CoV proteins in unexposed individuals was extensively described in Griffoni et al. (2020), without the specific identification of sequence targets. Other work describing correlations of CD4+/CD8+ T cell differential phenotypes between acute (highly activated cytotoxic) and convalescent (stem-like memory) patients was conducted by Karolinska COVID-19 Study Group (37). Interestingly, they described the occurrence of SARS-CoV-2-specific T cell responses elicited in the absence of circulating antibodies in non-infected individuals, suggesting that previous contacts could be the triggers of these cross-reactive events.

In this regard, given the previous identification of a similar target from the heterologous Influenza virus (M158-66 GILGFVFTL) with a SARS-CoV epitope, the next step was to scrutinize other trigger candidates on previously described viral epitopes. To perform the comparison, we recover pMHC structures on CrossTope T cell epitope databases (http://crosstope.com/ *)* searching for immunogenic fingerprints common to SARS-CoV-2 epitopes and unrelated viruses. The results from these comparisons were newsworthy. When we look for pHLA-A*02:01 structures, not only the previous example of M1_58-66_ IAV epitopes matched with SARS epitopes. Ten out of twenty CD8+ coronavirus epitopes, recovered from Immune Epitope Database, have counterparts in targets from common circulating viruses, concerning depicted molecular features. **Figure 3B** presents four examples of SARS-CoV peptides presenting stunning structural identity with unrelated viral epitopes. The matched targets belong to viruses from three different families (Herpesviridae, Poxviridae, and Flaviviridae), being important to show that these targets would probably not be investigated and selected in an approach using conventional methods, given that no apparent identity is presented by any of these structurally related epitopes with SARS-CoV sequences. The remaining comparisons can be viewed in **Figure S3**. Importantly, when we consider these image correspondences, the comparisons with pMHCs from unrelated viruses were more conspicuous than those from other representatives of HCoVs. It seems a paradox, given the natural expectation (considering its phylogenetics proximity) of a more intimate relation between alpha and beta CoVs peptides with SARS-CoV epitopes. In the future, maybe we need to change our prospection point of view to a more general search not only restricting it to phylogenetically related members.

### The impact of variants of concern on T cell epitope recognition

**Figure 4:**
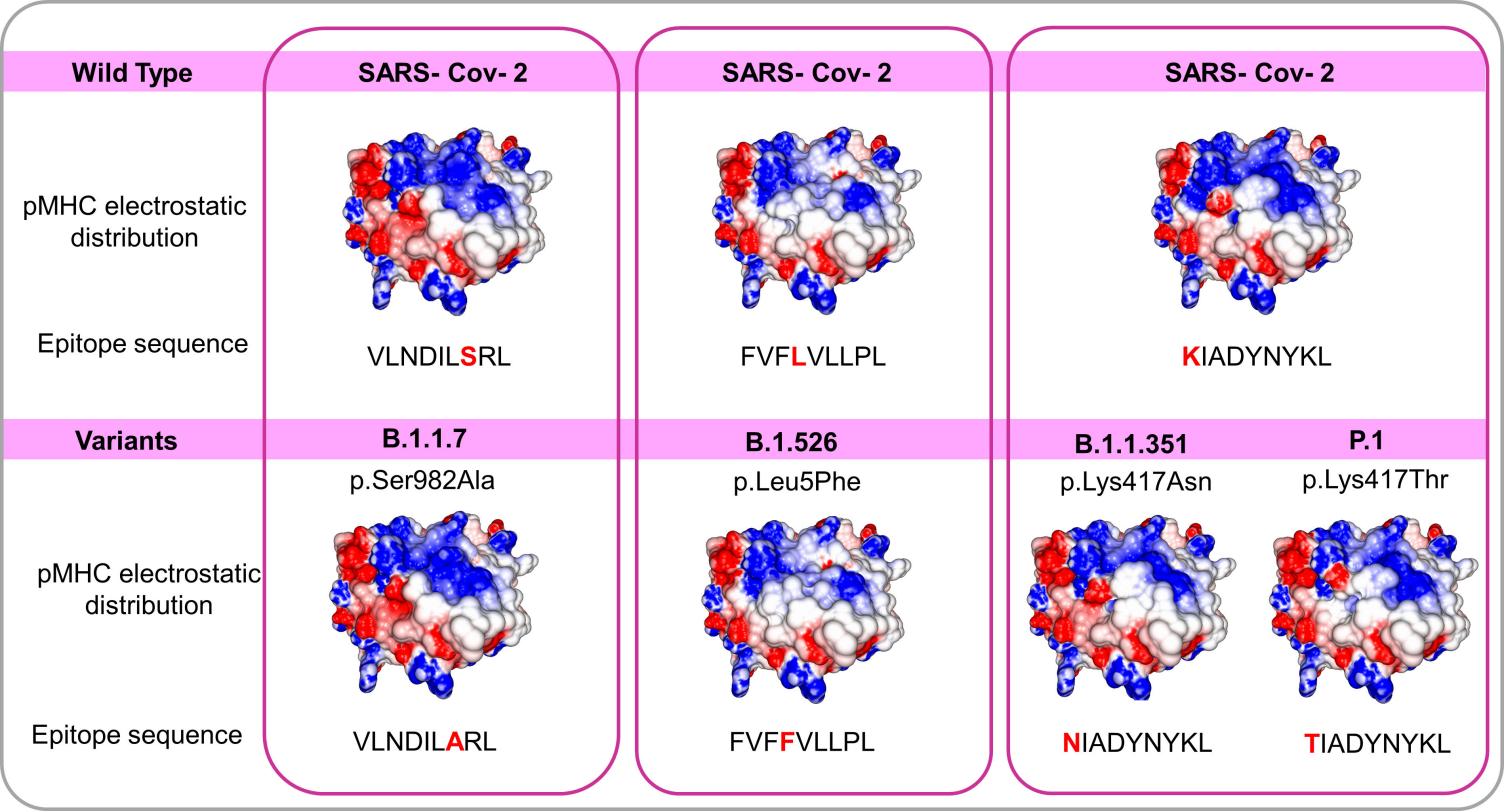
*Comparisons among pMHCs carrying wild SARS-CoV-2 epitopes and the correspondent variant sequences in SARS-CoV-2 Spike’ protein*. The electrostatic distributions surfaces from the wild type and correspondent variants do not differ, even presenting amino acid changes in peptides suggesting a preserved TCR recognition. The variants B.1.1.7 B.1.526 B.1.1.351 and P.1 with their respective changes (S982A L5F K417N and K417T respectively) and correspondent epitope sequences.

**Figure 5:**
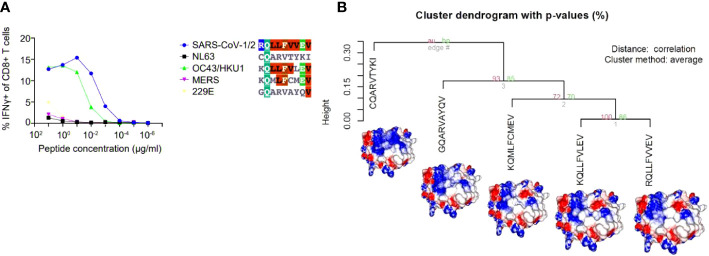
A hierarchical clusterization of pMHC’s electrostatic surfaces of different epitope sequences belonging to distinct coronaviruses (SARS-CoV-½, NL63, OC43/HKU1, MERS, 229E). In the chosen example, five sequences (in the context of HLA-A*02:01) from the analysed coronavirus were challenged against the same TCR and presented the pattern of recognition showed in **(A)**. In **(B)**, we depicted the HCA and images from the customized pMHC model complexes demonstrating that the epitopes that elicited the cross-reactive response were the most similar (from OC43/HKU-1 and SARS-CoV-1/2), providing an explanation for the observed pattern of cross-reaction. The figure presenting the results in A was extracted, and modified from (46).

The arising of Variants of Concern (VOC) became prevalent in the last months, increasing the number of infections and presenting a more pronounced contagious potential, which probably occurs due to their mutational landscape favoring an increased ACE-2 receptor affinity (38, 39). Some lineages such as B 1.1.7 were associated with the highest in-hospital mortality, which was 20% higher in the second wave of infection (40). Nevertheless, it was caused by an increased demand concomitant with a lack of structure to properly care for these patients, especially in developing countries. VOC infection provokes similar clinical manifestations as the wild-type strain, however, a cohort study from Challen (41) evidenced an increased virulence in the UK variant, which should be confirmed in studies conducted during warmer seasons to exclude this variable in the enhanced risk of death caused by this variant. Two recent works did not present an increase in the severity and number of the symptoms of deaths in patients infected with B.1.1.7 lineage (42, 43). A contributing aspect to be investigated refers to the initial inoculum, which could be amplified by the S protein gain of affinity, resulting in a viral load augmentation, with higher disease severity and death rates increment (44, 45). It seems that even higher infectivity is counterbalanced by a preserved cellular response. A recent study conducted by researchers of La Jolla Institute demonstrated that epitope mutations do not disrupt CD4+ and CD8+ T cell responses (25). The present work provides some examples of structural T cell recognition sustenance, which could explain the response maintenance in individuals infected by the ancient strain or those vaccinated with Pfizer and Moderna vaccines (tested in La Jolla Institute study), immunogens that stimulate endogenous production of antigen, facilitating its presentation to T cells. We investigated if some of the mutations presented by VOCs include the sequence positions of our analyzed epitopes. Four variants have amino acid alterations in immunogenic epitopes corresponding sequence regions (**Figure 4**). In all cases, there are no substantial physicochemical alterations that could be abolishing their TCR cross-recognition. It emphasizes the cross-protection potential of wild types and vaccines favoring an antigen processing pathway of their antigens against the VOCs. Substitutions in epitope viral strains are responsible for abolishing the response in the Hepatitis C virus. In this case, the mutations cause an alteration in the electrostatic potential distribution of wild-type and the variant strains (**Figure S4**), which was not observed in SARS-CoV-2 VOC examples. In an experiment performed by Nesterenko et al., 2021 (46) epitope homologs were identified and synthesized and T cell cross-reactivity was assessed *via* peptide titration assay. These different coronaviruses targets exhibited a diverse pattern of reactivity with different degrees of variation in the amino acid sequence, which could not be explained by sequence analysis. When we modeled the epitopes from different alpha and beta coronaviruses members, the cross-reactive peptides presented the more similar pMHC surfaces (OC43 and SARS-CoV-2) (**Figure 5**).

### Conclusion

In COVID-19, the aetiological agent, SARS-CoV-2, is well established. Nevertheless, the same cannot be deep-stated regarding all elements participating in severe acute respiratory syndrome (SARS) etiology. For this purpose, aspects of cellular response were approached: the structural analysis of immunogenic features presented by SARS-CoV-2, its variants, and unrelated viral epitopes. The epitope structural analysis and their relationship with other HCoVs and unrelated viral targets unveiled noteworthy observations. These pieces of evidence may open two avenues of investigation.

The high degree of molecular conservation between SARS-CoV-1 epitopes and their corresponding sequences in SARS-CoV-2 allows its use in vaccine development to stimulate cross-reactive responses, covering distinct SARS-CoV-2 strains, for example. In this regard, animal reservoirs should be inspected looking for beta CoVs with the potential to spill out of their natural hosts to humans. Peptides sequences from these putative HCoVs pathogens could be structurally compared searching for cross-reactive T cell targets to be used in a virtual future occurrence of a new coronavirus spillover phenomenon. Such preventive strategies could abbreviate steps to develop immunotherapeutic methods, avoiding the emergence of new pandemics. The second line of the investigation resulted in an even more attractive hypothesis. Previous infections with different alpha/beta-CoVs and unrelated common viruses can be generating memory T cells against SARS-CoV-2 in a significant portion of the population. This pool of cells in different individuals could be providing a universal immunogenic shield against SARS-CoV-2 and, probably, against other potentially emergent and endemic viruses. This mechanism seems to be evolutionarily constructed by regular cross-reactive contacts. Moreover, the defense appears to be associated with prevalent alleles, which probably present peptides harboring fingerprints of immunogenicity shared by epitopes that regularly infect humans. The last investigated point was the VOIs substitutions present in immunogenic regions of SARS-COV-2 epitopes. We observed that the mutations do not alter the TCR interaction region of pMHCs. In this way, it seems that the CD4+ and CD8+ responses against these targets are preserved, where the elicited T cells responses seem to be directed toward constrained regions of these viruses.

## Methods and resources

### HLA-A*02:01 allele frequencies among the countries

The HLA-A*02:01 distribution and its frequencies for all included countries were obtained from The Allele Frequency Net Database (47). The applied filter includes only gold and silver standard population samples, considering n>50 and random studies. Weighted frequencies were calculated based on all populations’ frequency information for the resulting 57 countries. **Table S1** presents the frequencies and sample sizes (total population) for each country. The dplyr and ggplot2 packages in the RStudio evaluate the distribution of data, represented in **Figure 1**.

### SARS-CoV Epitopes prospections

The Immune Epitope Database and Analysis Resource (IEDB - https://www.iedb.org/) (48) was accessed to prospect and recover experimental coronavirus epitopes (the search was refined through the SARS-CoV-1 organism, T cell positive assays, and MHC allele), resulting in 20 immunogenic peptides restricted to HLA-A*02:01 allele (**Table 1** compiles its Epitope IDs). Those epitopes derived from the N (nucleocapsid) protein, Surface (spike) protein, and Membrane glycoprotein. Aiming to check if they have a counterpart in the SARS-CoV-2 proteome, we perform a Needleman-Wunsch Global Align Nucleotide Sequences (BLAST) using the protein sequences from 2002/2003 SARS virus against SARS-CoV-2 proteome sequence from Wuhan ancient genome. NetMHCcons tool (49) was applied to predict binding affinity in the discordant SARS-CoV-2 sequences and also for other HCoVs peptides. The 229E and NL63 Alphacoronaviruses plus the OC43 Betacoronavirus strains were also checked for corresponding SARS-CoV epitopes sequences, screening their protein correspondences searching for some amino acid identity with immunogenic targets described for SARS-CoV-1.

### Variants prospection

The panel of amino acids reported changes in the spike (S) protein in SARS-CoV-2 VOCs (50–52) were screened to evaluated if they fall in the HLA-A*02:01 epitopes included in our analysis. Four changes were matched to three epitopes: B.1.526 (p.Leu5Phe) to epitope FVF**L**VLLPL, B.1.1.351 (p.Lys417Asn) and P.1 (p.Lys417Thr) to epitope **K**IADYNYKL, and B.1.1.7 (p.Ser382Ala) variant occur in the epitope VLNDIL**S**RL already described in **Table 1**. Spike protein sequences were verified in UniProt code: P0DTC2 (SPIKE_SARS2).

### Structural analysis

All the recovered epitopes for Alpha and Beta Coronaviruses and analysed VOCs were modeled in HLA-A*02:01 context to investigate their TCR interaction surfaces looking for immunogenicity fingerprints and shared structural features. The full applied rationale can be found in (53). The Docktope tool (http://tools.iedb.org/docktope/) allowed to produce pMHC models containing the analysed peptides anchored to the HLA-A*02:01 Class I MHC (pMHC). This tool allowed the generation of models based on a D1-EM-D2 approach, involving a first step of molecular docking (D1) performed using Autodock Vina program between the MHC-I and the epitope provided; followed by an energy minimization step (EM) to correct possible steric clashes between the epitope and the MHC-I; and a second round of molecular docking (D2) aiming to refine the structure.

The generated tridimensional structures were inputted in GRASP to calculate their electrostatic surfaces, verifying amino acid changes impact on the overall charge distribution in those models. The GRASP aplication allowed to generate colors according the charge distribution considering the model surface: the eletrostatic potential ranges from -5 (negative charges in red) to 5 (positive charges in blue) passing through neutral (white color). To quantify this investigation, we utilize the software ImageJ to analyze the RGB content of several spots in the pMHC structure where contact with the TCR tends to occur and save this data in the form of mean, mode, and standard deviation in a numeric form. The numeric RGB data is then clusterized utilizing the R software package Pvclust to perform the hierarchical clusterization based on the data provided and separate different structures from each other while clustering similar structures based on their electric charge distribution.

Images generated for those complexes (pMHC) were compared against all structures included at the CrossTope Structural Databank, looking for shared patterns which may indicate cross reactivity among structurally similar epitopes. The CrossTope database contain pMHC images of distinct immunogenic epitopes described in the literature for prevalent human alleles. The dataset images were analyse utilizing an electrostatic range which varies from -5 to 5.

## Data availability statement

The original contributions presented in the study are included in the article/**Supplementary Material**. Further inquiries can be directed to the corresponding author.

## Authors contributions

EA: Visual and Statistical Analysis, Investigation, Formal analysis. MB: Formal analysis, Resources, MM: Data curation, Investigation, Writing. GV: Conceptualization, Writing, Supervision, Project administration. All authors contributed to the article and approved the submitted version.

## Funding

This work was supported by scholarships from National Council for Scientific and Technological Development (CNPq); and National Council for Improvement of Higher Education (CAPES).

## Acknowledgments

We thank the scholarships from National Council for Scientific and Technological Development (CNPq) and National Council for the Improvement of Higher Education (CAPES) for their support.

## Conflict of interest

The authors declare that the research was conducted in the absence of any commercial or financial relationships that could be construed as a potential conflict of interest.

## Publisher’s note

All claims expressed in this article are solely those of the authors and do not necessarily represent those of their affiliated organizations, or those of the publisher, the editors and the reviewers. Any product that may be evaluated in this article, or claim that may be made by its manufacturer, is not guaranteed or endorsed by the publisher.

## Supplementary material

Supplementary Figure 1 Comparison of epitope sequences and pMHC electrostatic distribution among SARS-1 and SARS-2. Five out of 20 paired peptides show divergent sequences. Nevertheless, the presence of amino acid changes does not cause alterations in the pMHC electrostatic distribution pattern. This immunogenicity predicted by the sharing of structural features was already experimentally confirmed, considering that all corresponding SARS-CoV-2 stimulates cytotoxic responses. pMHC electrostatic distribution is colored as follows: from -5 (electronegative in red) to +5 (positive in blue) passing through 0 (neutral white). Divergent amino acids detached in bold.

Supplementary Figure 2
*TCR interacting surfaces from pMHC immunogenic structures.* Nine pMHC from HLA-A*02:01 bound to experimental epitopes. We provided this panel to demonstrate the molecular diversity among immunogenic targets.

Supplementary Figure 3
*Additional comparisons of SARS-CoV epitopes and other immunogenic epitopes from the CrossTope Database.* Shared fingerprints in the above-exemplified pairs of structures, indicating that cross-reactivity non-related triggers can be a widespread phenomenon.

Supplementary Figure 4
*Two distinct Hepatitis C virus epitopes and their pMHCs electrostatic patterns.* The variation (detached in red) between the wild-type peptide sequence (CVNGVCWTV) with its variant peptide (TVGDVMWTV) leads to divergent charge distribution at the indicated pMHC surface area (pink square). This variant peptide, from HCV genotype 3, aborts the cellular response in lymphocytes that recognizes the WT peptide. The electrostatic potential distribution is colored as follows: from -5 (electronegative in red) to +5 (positive in blue), passing through 0 (neutral in white).
