# Supplementary figures and images for: Viral immunogenic footprints conferring T cell cross-protection to SARS-CoV-2 and its variants
Source: Front Immunol. 2022 Jul 28;13:931372. doi: 10.3389/fimmu.2022.931372 (PMC9366040; doi:10.3389/fimmu.2022.931372)

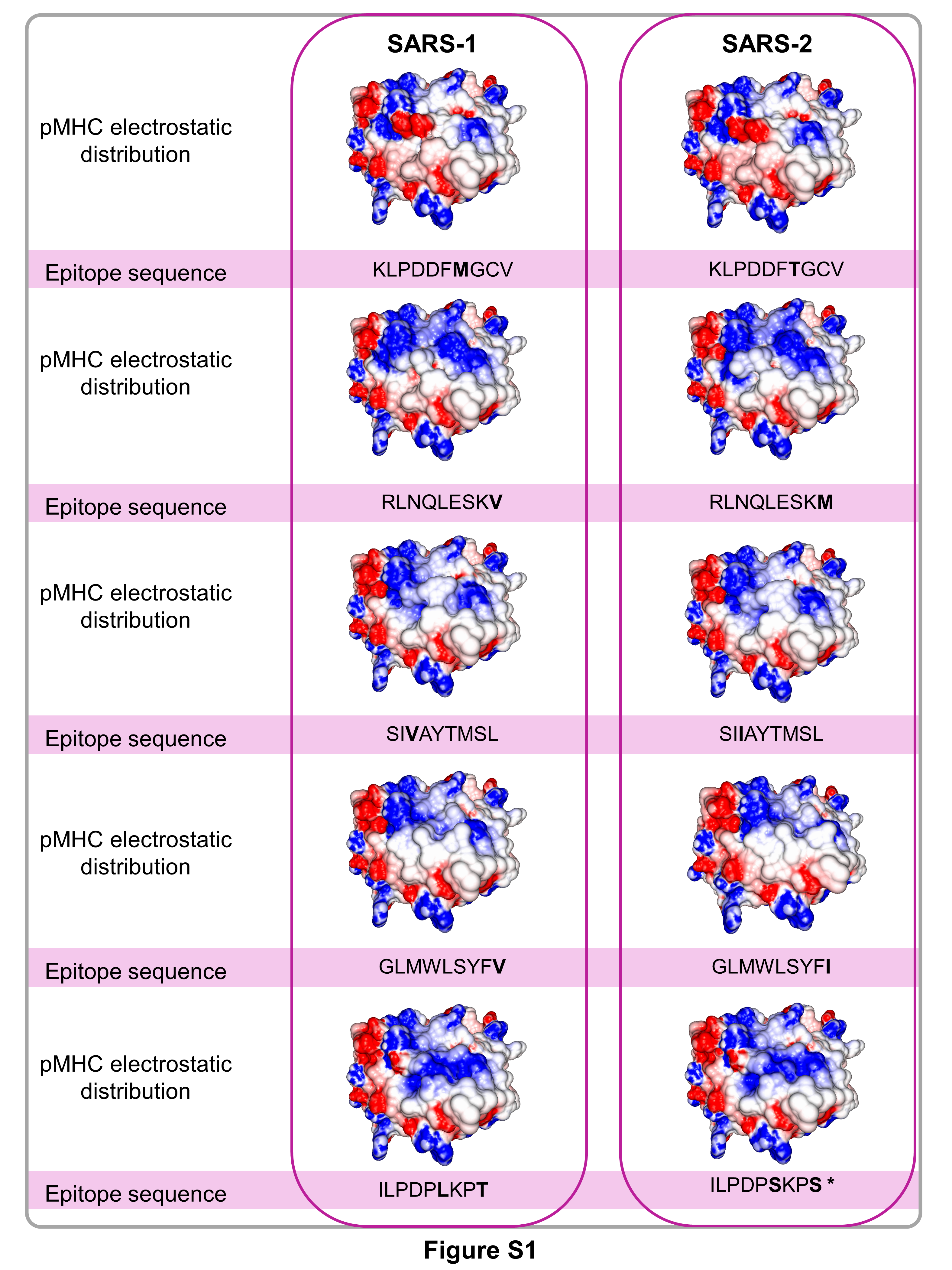

Supplement: Supplementary Figure 1 — Comparison of epitope sequences and pMHC electrostatic distribution among SARS-1 and SARS-2. Five out of 20 paired peptides show divergent sequences. Nevertheless, the presence of amino acid changes does not cause alterations in the pMHC electrostatic distribution pattern. This immunogenicity predicted by the sharing of structural features was already experimentally confirmed, considering that all corresponding SARS-CoV-2 stimulates cytotoxic responses. pMHC electrostatic distribution is colored as follows: from -5 (electronegative in red) to +5 (positive in blue) passing through 0 (neutral white). Divergent amino acids detached in bold. [file Image_1.tif]

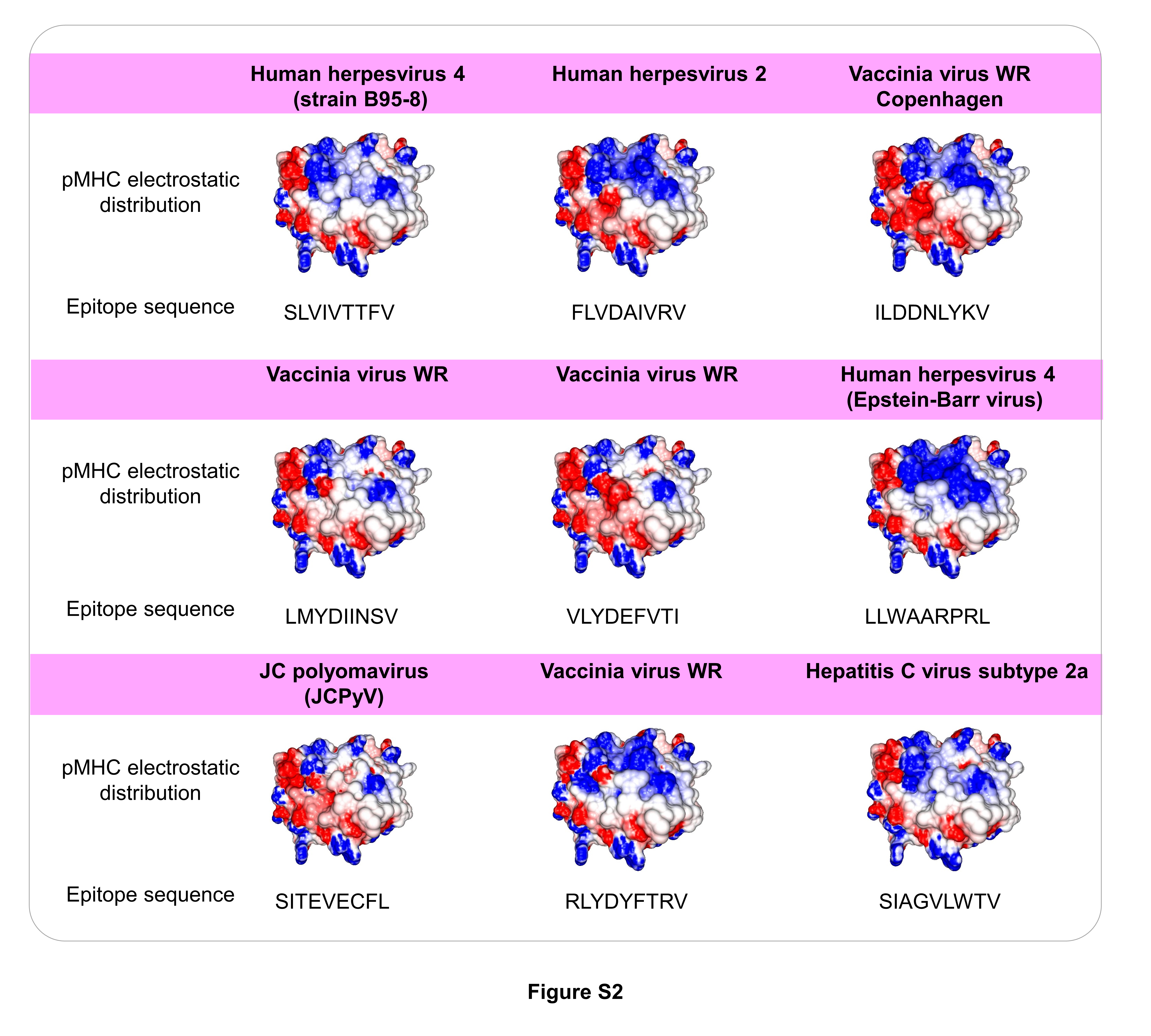

Supplement: Supplementary Figure 2 — TCR interacting surfaces from pMHC immunogenic structures. Nine pMHC from HLA-A*02:01 bound to experimental epitopes. We provided this panel to demonstrate the molecular diversity among immunogenic targets. [file Image_2.tif]

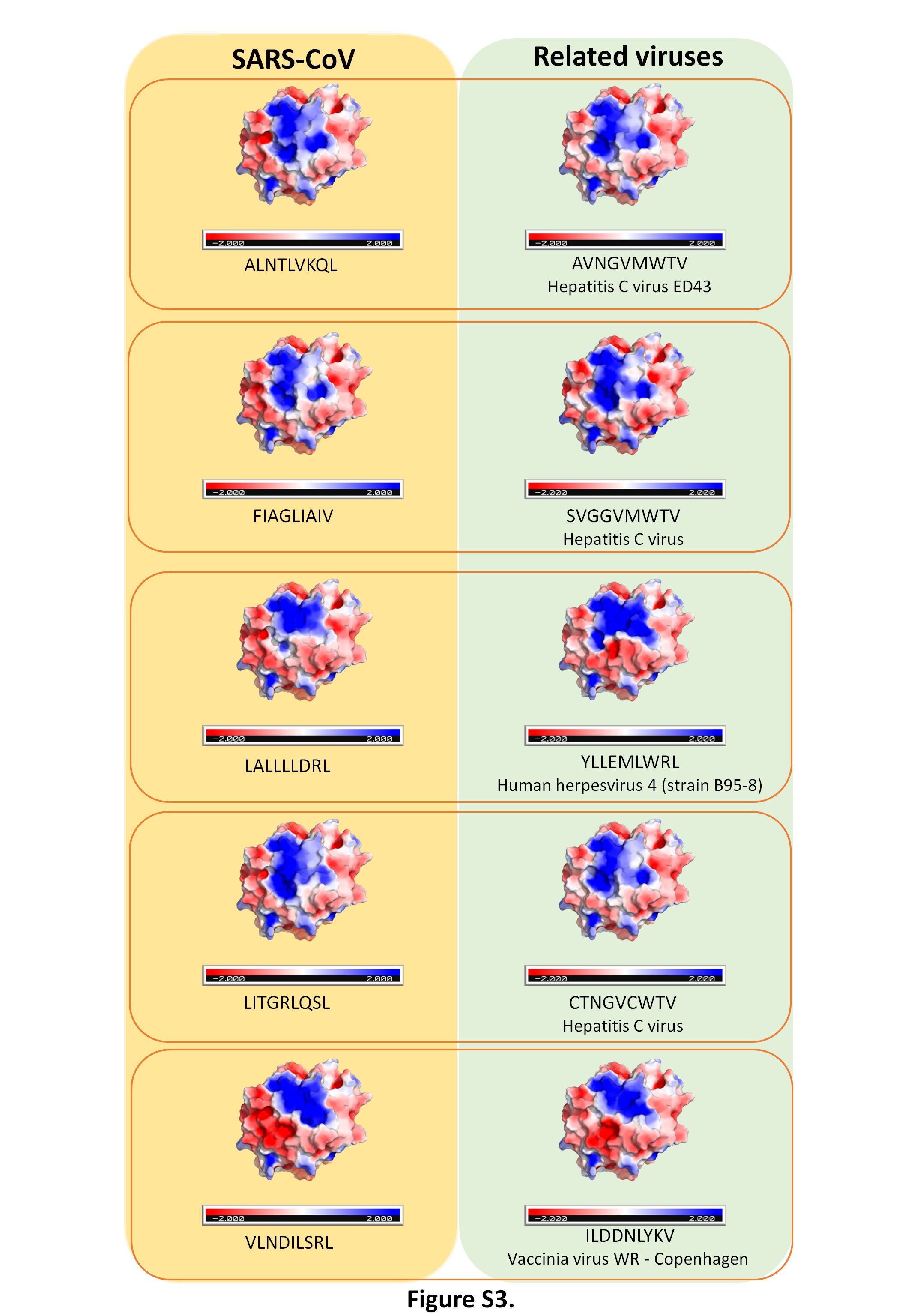

Supplement: Supplementary Figure 3 — Additional comparisons of SARS-CoV epitopes and other immunogenic epitopes from the CrossTope Database. Shared fingerprints in the above-exemplified pairs of structures, indicating that cross-reactivity non-related triggers can be a widespread phenomenon. [file Image_3.tif]

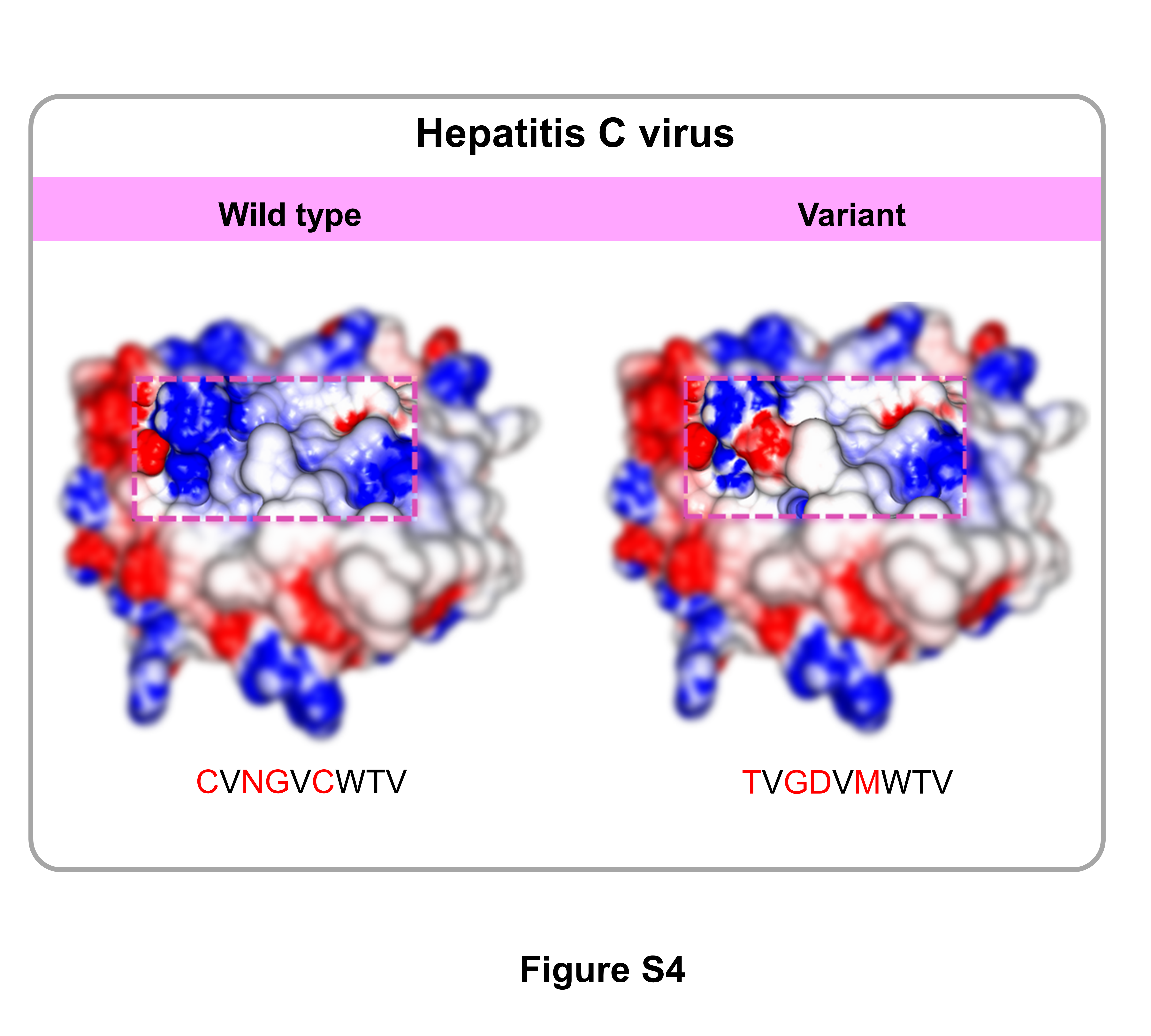

Supplement: Supplementary Figure 4 — Two distinct Hepatitis C virus epitopes and their pMHCs electrostatic patterns. The variation (detached in red) between the wild-type peptide sequence (CVNGVCWTV) with its variant peptide (TVGDVMWTV) leads to divergent charge distribution at the indicated pMHC surface area (pink square). This variant peptide, from HCV genotype 3, aborts the cellular response in lymphocytes that recognizes the WT peptide. The electrostatic potential distribution is colored as follows: from -5 (electronegative in red) to +5 (positive in blue), passing through 0 (neutral in white). [file Image_4.tif]
