# Supplementary material for: Viral immunogenic footprints conferring T cell cross-protection to SARS-CoV-2 and its variants
Source: Front Immunol. 2022 Jul 28;13:931372. doi: 10.3389/fimmu.2022.931372 (PMC9366040; doi:10.3389/fimmu.2022.931372)
Supplement: Supplementary file 5 [file Table_1.docx]

**Table S1. Countries data concerning HLA-A*02:01 frequencies and populational information**

| **Countries^A^** | **Sample size^B^** | **Frequency**  **(A*02:01)^C^** | **Death per million^D^** | **Cases per million^D^** | **Death/cases per million** | **Countries** | **Sample size** | **Frequency**  **(A*02:01)** | **Death/million** | **Cases/million** | **Death/cases per million** |
| --- | --- | --- | --- | --- | --- | --- | --- | --- | --- | --- | --- |
| **Argentina** | 135 | 20.071 | 1.244 | 53.349 | 0.023318 | **New Zealand** | 199 | 7.5452 | 5 | 506 | 0.009881 |
| **Armenia** | 100 | 15.5 | 1.229 | 67.026 | 0.018336 | **Nicaragua** | 339 | 17.66 | 27 | 1.006 | 0.026839 |
| **Australia** | 544 | 16.022 | 35 | 1.147 | 0.030514 | **Oman** | 118 | 21.6 | 333 | 32.024 | 0.010398 |
| **Brazil** | 263 | 21.19 | 1.579 | 61.326 | 0.025748 | **Pakistan** | 430 | 9.2744 | 67 | 3.124 | 0.021447 |
| **Cameroon** | 289 | 9.1744 | 31 | 2.120 | 0.014623 | **Peru** | 210 | 50.0 | 1.603 | 47.975 | 0.033413 |
| **Chile** | 87 | 19.271 | 1.237 | 57.207 | 0.021623 | **Philippines** | 50 | 30.0 | 127 | 7.400 | 0.017162 |
| **China** | 6476 | 11.3006 | 3 | 63 | 0.047619 | **Poland** | 20.653 | 28.49 | 1.473 | 65.361 | 0.022536 |
| **Colombia** | 1562 | 15.652 | 1.258 | 48.117 | 0.026145 | **Portugal** | 275 | 24.807 | 1.660 | 81.093 | 0.02047 |
| **Croatia** | 154 | 26.374 | 1.505 | 69.153 | 0.021763 | **Romania** | 348 | 25.0 | 1.283 | 51.654 | 0.024838 |
| **Cuba** | 112 | 17.9 | 39 | 7.296 | 0.005345 | **Russia** | 1.348 | 27.166 | 695 | 31.553 | 0.022026 |
| **Ecuador** | 63 | 25.4 | 953 | 19.029 | 0.050081 | **Sao Tome Island** | 98 | 10.678 | 157 | 10.146 | 0.015474 |
| **Finland** | 91 | 34.4 | 155 | 14.496 | 0.010693 | **Saudi Arabia** | 213 | 19.0 | 191 | 11.212 | 0.017035 |
| **Georgia** | 220 | 24.274 | 964 | 71.711 | 0.013443 | **Senegal** | 165 | 8.0132 | 63 | 2.293 | 0.027475 |
| **Germany** | 69.865 | 26.224 | 928 | 34.723 | 0.026726 | **Singapore** | 797 | 8.0132 | 5 | 10.289 | 0.000486 |
| **Ghana** | 131 | 10.69 | 24 | 2.883 | 0.008325 | **South Afrika** | 151 | 1.9934 | 886 | 25.934 | 0.034164 |
| **Greece** | 325 | 27.384 | 829 | 27.447 | 0.030204 | **South Korea** | 4.613 | 15.587 | 34 | 2.084 | 0.016315 |
| **Guinea Bissau** | 127 | 10.224 | 33 | 1.534 | 0.021512 | **Spain** | 4.335 | 23.68 | 1.626 | 71.134 | 0.022858 |
| **Hong Kong** | 12.056 | 18.233 | 27 | 1.530 | 0.017647 | **Sri Lanka** | 815 | 3.5859 | 27 | 4.372 | 0.006176 |
| **Indonesia** | 236 | 6.6 | 153 | 5.612 | 0.027263 | **Sudan** | 200 | 17.3 | 46 | 675 | 0.068148 |
| **Iran** | 124 | 11.674 | 751 | 23.397 | 0.032098 | **Sweden** | 284 | 24.699 | 1.338 | 82.958 | 0.016129 |
| **Ireland** | 251 | 24.611 | 949 | 48.059 | 0.019747 | **Taiwan** | 2.746 | 8.8763 | 0 4 | 44 | 0.009091 |
| **Israel** | 195.052 | 10.936 | 681 | 90.802 | 0.0075 | **Tunisia** | 195 | 16.667 | 759 | 22.081 | 0.034373 |
| **Italy** | 1153 | 22.853 | 1.861 | 61.271 | 0.030373 | **Turkey** | 142 | 25.7 | 384 | 42.093 | 0.009123 |
| **Japan** | 20.160 | 11.632 | 74 | 3.880 | 0.019072 | **Uganda** | 336 | 14.754 | 7 | 876 | 0.007991 |
| **Kenya** | 605 | 12.115 | 42 | 2.586 | 0.016241 | **USA** | 2.881.506 | 20.137 | 1.715 | 94.991 | 0.018054 |
| **Malaysia** | 1.684 | 8.2041 | 40 | 10.846 | 0.003688 | **Venezuela** | 55 | 21.6 | 60 | 5.959 | 0.010069 |
| **Mali** | 138 | 8.3 | 19 | 520 | 0.036538 | **Vietnam** | 170 | 2.1 | 0 4 | 27 | 0.014815 |
| **Mexico** | 1.436 | 23.775 | 1.577 | 17.361 | 0.090836 | **Zimbabwe** | 230 | 9.1 | 104 | 2.460 | 0.042276 |
| **Netherlands** | 1.474 | 27.65 | 971 | 76.868 | 0.012632 | **New Zealand** | 199 | 7.5452 | 5 | 506 | 0.009881 |

**^A^**The included countries were selected by the availability of HLA-A*02:01 frequencies in The Allele Frequency Net Database gold and silver standard. ^B^ Sample sizes were estimated by the sum of countries’ populations values below 50 were discarded.

^c^HLA-A*02:01 frequencies values correspond to a weighted average of all available populations in each country following the selection criteria

^D^Information about death and cases per million were obtained from COVID-19 Dashboard by the Center for Systems Science and Engineering (CSSE) at Johns Hopkins University (*recovered on April 7th 2021)* and used to estimate death/case per million.
